# Individual heterogeneity, educational attainment and cardiovascular mortality: a pooled analysis of Norwegian health surveys

**DOI:** 10.1136/bmjph-2023-000104

**Published:** 2024-09-23

**Authors:** Huong Thu Nguyen, Tron Anders Moger, Morten Valberg, Eirik Degerud, Christian M Page, Marissa LeBlanc, Øyvind Næss

**Affiliations:** 1 Department of Community Medicine and Global Health, University of Oslo, Oslo, Norway; 2 Centre for Fertility and Health, Norwegian Institute of Public Health, Oslo, Norway; 3 Department of Health Management and Health Economics, University of Oslo Faculty of Medicine, Oslo, Norway; 4 Oslo Centre for Biostatistics and Epidemiology, Oslo University Hospital, Oslo, Norway; 5 National Institute of Occupational Health, Oslo, Norway; 6 Section for Statistics, Department of Mathematics, University of Oslo, Oslo, Norway; 7 Division of Infection Control, Norwegian Institute of Public Health, Oslo, Norway; 8 Oslo Centre for Biostatistics and Epidemiology, University of Oslo, Oslo, Norway; 9 Norwegian Institute of Public Health, Oslo, Norway

**Keywords:** Public Health, Community Health, Epidemiology

## Abstract

**Background:**

Smoking, physical inactivity, cholesterol level and systolic blood pressure are well-established risk factors for cardiovascular diseases (CVD). However, even among individuals with similar levels of these factors, a substantial degree of variation in risk may still remain. We investigated the variation in this unexplained risk across educational levels.

**Methods:**

The study population (N=451 800) was from Norwegian health surveys and linked to the National Education Database and the Norwegian Cause of Death Registry. We used survival analysis with frailty models to measure unobserved heterogeneity (frailty variation). Models were stratified by three educational levels. We highlight the degree of heterogeneity by presenting Gini coefficients and indicate how much the unobserved heterogeneity differed across levels of education from the estimated parameters of the frailty distributions. Lorenz curves were plotted for a graphical representation of inequalities in individual risk.

**Main results:**

The estimated frailty variances were 24.96, 34.12 and 42.37 in the low, middle and high education groups, respectively, before adjusting for risk factors and 3.76, 7.12 and 7.82 after adjusting for risk factors. The corresponding Gini coefficients were 0.75, 0.84 and 0.86 for low, middle and high education groups, respectively.

**Conclusion:**

A large share of the variation in CVD mortality was explained by the observed risk factors. However, a substantial individual unobserved variation in CVD mortality remained after adjusting for these risk factors. Our findings indicate that the unobserved variation in CVD mortality could vary somewhat between educational groups, but that education does not explain the major share of the remaining substantial heterogeneity.

**WHAT IS ALREADY KNOWN ON THIS TOPIC**

- Not everyone with the same level of risk factors develops cardiovascular disease (CVD).
- Unmeasured factors may play a role in the risk and vary systematically across educational levels.

**WHAT THIS STUDY ADDS**

- We extracted information on CVD risk factors from Norwegian health surveys, the highest educational attainment from the National Education Registry and data linked to the Cause of Death Registry, with participants categorised into three levels of education (low, middle and high).
- The results indicate that the size of unobserved heterogeneity in risk of dying from CVD (frailty variation) could differ somewhat by length of educational attainment even if they had similar levels of CVD risk factors; however, a substantial unobserved individual heterogeneity in CVD mortality remains.

**HOW THIS STUDY MIGHT AFFECT RESEARCH, PRACTICE OR POLICY**

- A large part of the variation in CVD mortality was explained by established risk factors; however, a substantial variation remained both in the full sample and across educational groups.
- Preventive strategies may need to consider the potential importance of underlying unobserved individual factors in subgroups.

## Introduction

 Cardiovascular disease (CVD) mortality has decreased in many high-income countries in recent decades. At the population level, secular declines and differences in CVD mortality between population groups have, in addition to improvements in medical treatment,^1^ been attributed to improvements in a few established risk factors, such as tobacco smoking, physical inactivity, total cholesterol levels and high systolic blood pressure.^2^ Meanwhile, socioeconomic inequalities have persisted in CVD and other non-communicable diseases. This has been observed while effective medical treatment and ambitious preventive programmes have been introduced targeting to reach all population groups.^3 4^

Although CVD mortality has declined in all social groups due to reduced CVD risk factors, healthier lifestyle, better living standards and technological advancements in diagnostics and treatment, the excess burden due to socioeconomic inequalities is substantial and persists across modern welfare states providing publicly funded healthcare and social security benefits to its inhabitants.^4^ It has been suggested that underlying individual factors may have become more important for the health inequalities in modern welfare states and several explanations may be important. First, intergenerational social mobility in many high-income countries has resulted in social groups becoming more heterogeneous with respect to individual differences, especially in education.^5^ Second, couples tend to mate increasingly based on similar educational and cognitive background.^6^ Third, with increased availability of energy-dense foods in modern societies and passive transportation, individuals are left with more responsibility in terms of healthy lifestyle choices.

Some of these underlying factors may be measured, such as cognitive ability.^7 8^ Others are difficult to measure but may still vary systematically between social groups. The influence of these factors on the risk of diseases may be more subtle than baseline measurements of risk factors alone can estimate, as they may capture the lifelong effect of risk factors and possibly explain more of the social gradient.^9^,^11^ This individual variation may differ between socioeconomic groups, and better knowledge is important from a public health policy point of view because general preventive measures against CVD may increase inequalities if they rely too much on individual resources, such as cognitive or other non-cognitive skills.

We aimed to quantify how much these risk factors account for future risk of CVD mortality as well as how much risk they do not account for, referred to as unexplained variation, by using frailty models in survival models.^12^ The frailty parameter can be considered as unobserved heterogeneity because it represents an estimate of variation that has not been explained by measured covariates included in the frailty model.^13^ The literature on frailty models, particularly in recent reviews, offers insightful perspectives on their application and development. Balan and Putter^14^ contributed to the understanding of these models, emphasising their significance and utility in statistical analyses. Rubio *et al*^15^ applied frailty models to address individual heterogeneity arising from factors such as smoking habits. The authors also provided a review on the use of frailty models, highlighting their importance in capturing variations in survival data that standard models might not adequately address.^15^ In our context, by fitting a frailty model that includes established CVD risk factors, the variation estimated by a frailty parameter may indicate differences in risk between individuals that go beyond measured cardiovascular risk factors, thus measuring individual heterogeneity.^12^ We investigated if the variation in the additional unexplained CVD mortality risk differs systematically across levels of education.

## Methods

### Study population, data linkage and selection

Our study population included participants from three Norwegian health examination studies: the Cohort of Norway (CONOR),^16^ the Age 40 Programme (A40P)^17^ and the Counties Study (NCS),^18^ with overall participation rates of 58%, 70% and 86%, respectively. The CONOR is a collection of several regional Norwegian cohort studies performed in 1994–2003, where both men and women at various age groups were interviewed. The A40P collected cardiovascular health surveys in 1985–1999 that invited men and women aged 40–42 years to participate. The NCS included three cardiovascular screenings in the counties of Oppland, Sogn og Fjordane and Finnmark in 1974–1988.

Our study sample initially included 684 156 observations nested in 565 564 individuals. Only data from one survey per individual were selected. For participants with data from more than one survey, information from those who attended the CONOR was selected. If they did not attend the CONOR, information from the NCS was used. If they did not attend the CONOR or the NCS, we included participants from the A40P study. 118 592 overlapping observations were excluded (online supplemental figure 1). In recent decades, a larger share of the Norwegian population finished higher education.^19^ This means that similar level of educational attainment may not represent the same underlying risk among those who were young in the 1960s compared with later. For this reason, we chose to focus on a narrow age band, that is, 35–65. This also avoids including risk factor assessment among the youngest and the oldest of the participants as the effect on CVD in these groups may differ from people in middle age. Missing data on important variables were excluded by using listwise deletion. The final sample size after duplicates and missing variable were removed was 451 800.

### Main exposure and covariates

We retrieved data on education from the National Education Registry and recoded and divided the participants into three levels: low (primary and secondary schools), middle (from high school to university) and high (from university to doctoral degree or higher). More specifically, the National Education Database provided data on the highest level of education obtained until 2011 (1–8, where 1 equals primary school and 8 a master or doctoral degree). We recoded and divided the participants into three educational groups, namely low level (1– 2), middle level (3–5) and high level (6–8). Specifically, low level covers up to 10 years of schooling (primary and lower secondary), middle level includes tertiary vocational education and high level encompasses undergraduate and postgraduate studies.

For each survey, we extracted information on smoking and classified the participants into non-current smokers and current smokers. Since smoking is considered a common CVD risk factor and an important predictor of individual risk, we obtained information from four screenings in the NCS. Data on smoking status were derived from one of the four screenings for each individual. In cases where a participant was identified as a current smoker in one screening but not in others, they were classified as current smokers. Physical activity was harmonised into a four-graded scale from sedentary (1) to hard physical (4). We obtained information on other established CVD risk factors, including body mass index (kg/m^2^), total cholesterol (mmol/L) and systolic blood pressure (mm Hg), from objective measurements performed by survey personnel.

### Outcome data and follow-up

Participants were linked to the Cause of Death Registry and followed until death from any cause, emigration or 31 December 2020. This provided outcome data on the causes of death using the ninth and tenth revision of the International Classification of Diseases (ICD). The primary outcome was CVD mortality (1990–1995: ICD-9: 390–459; 1996–2014: ICD-10: I00–I99). The information was almost exclusively based on certificates filled out by on-site medical doctors.

### Statistical methods

We considered three subgroups of the final population with 451 800 individuals as three samples corresponding to three levels of education: low (n=96 872), middle (n=236 929) and high (n=117 999). The outcome is CVD mortality. The age of 35 was chosen as the start of follow-up. First, we implemented parametric survival models with a Weibull baseline. We fitted the models with only two covariates: sex and one of the risk factors (current smoking, physical activity, body mass index, total cholesterol, systolic blood pressure). We then fitted the parametric survival models including all these CVD risk factors with and without gamma distributed frailty. Second, for each educational level *i* (*i*=1,2,3), we estimated a frailty model. Frailty models allowed us to examine unobserved heterogeneity that may not be explained by the covariates in the model. The covariates contained sex and the established CVD risk factors, including smoking status, physical activity, body mass index, total cholesterol and systolic blood pressure. As a result, our frailty models can be given by the following:

$$\begin{array}{l} h_{i}\left(t|Z_{i}\right)\;={\text{Z}}_{i}\text{p}{\text{t}}^{p-1}\exp\left(\beta_{0}+\beta_{1}x_{\text{i1}}+\beta_{2}x_{\text{i2}}+\cdots +\beta_{j}x_{\text{ij}}+\cdots +\beta_{6}x_{\text{i6}}\right) \end{array}$$

where *x*_ij_, j=1,…,6, are covariates. A separate model was fitted for each educational group. Analysis time was measured in years (number of days divided by 365.25) of follow-up from the date the individual turns 35 years until the date of death or censoring. The variability in Z*i* determined the degree of heterogeneity between individuals in a given educational group.

We assumed that the baseline hazard function followed a Weibull distribution with baseline hazard function^20^: h_0_(t)=pt^p−1^. The shape parameter, p, allowed the density to take a variety of shapes. The shape may vary according to the level of education. Note that $exp(\beta_{0})$ corresponds to the scale parameter of the Weibull baseline hazard rate. We assumed a gamma distributed frailty with parameter θ*,* which is one of the most widely used in frailty analysis.^21^ Despite the choice of a narrow age range of 35–65 years at participation, it seems that the lower educated group had slightly higher age and greater age dispersion. We performed sensitivity analysis including only the study participants who were part of the A40P. They have homogenous age range compared with other health surveys. We also performed a sensitivity analysis in an unstratified model and included educational attainment as covariate in addition to risk factors.

The Gini index and Lorenz curve are standard tools in economics used to express inequalities between individuals in a population.^22^ Here we calculated the Gini coefficients for the frailty distributions in the three educational groups using the acid package in R.^22^ The Gini coefficients represented inequality in frailty for different education strata. A Gini coefficient of 0 expressed perfect equality, that is, no unobserved variation between individuals. A Gini coefficient of 1 indicated maximal inequality. Lorenz curves were plotted to provide a visualisation of inequalities in individual risk according to educational level. The estimation of frailty models was conducted using STATA V.14 software.

### Patient and public involvement

No patient and public involvement.

## Results

The mean (SD) age at the time of survey participation was 42.6 (5.3) years. 52.2% were women. The prevalence of low, middle and high level of education was 21.44%, 52.44% and 26.12%, respectively (table 1). People with low level of education (n=96 872) were more often female and had higher prevalence of the established CVD risk factors. Those with high level of education (n=117 999) had the lowest prevalence of CVD risk factors. Participants who were excluded for missing values tended to be older (online supplemental table 1). In the study, the low education group was slightly older, with an average age of 43.71 years and a higher age dispersion (SD 6.81), compared with the middle education group (average age 42.41 years, SD 4.94) and the high education group (average age 42.09 years, SD 4.25). Compared with other groups, the group with high level of education had lower proportion of current smokers, were more physically active, and had lower levels of body mass index, total cholesterol and systolic blood pressure on average. Overall, 11 932 participants died from CVD, accounting for approximately 2.6% of the sample. CVD mortality rates varied significantly by educational level: 4.83% in the low education group, 2.43% in the middle education group and 1.27% in the high education group (table 1).

**Table 1:** Descriptive statistics according to categories of education among participants in the Norwegian health surveys in the 35–65 years age range (N=451 800)

	Educational level			All
	Low	Middle	High	
All (%)	96 872 (21.44)	236 929 (52.44)	117 999 (26.12)	451 800 (100)
All-cause deaths (%)	18 315 (18.91)	26 294 (11.10)	8176 (6.93)	52 785 (11.68)
CVD deaths (%)	4675 (4.83)	5754 (2.43)	1503 (1.27)	11 932 (2.64)
Age (years)	43.71 (6.81)	42.41 (4.94)	42.09 (4.25)	42.60 (5.27)
Sex (male) (%)	42 630 (44.01)	116 587 (49.21)	56 640 (48.00)	215 857 (47.78)
Current smoker (yes) (%)	51 359 (53.02)	97 258 (41.05)	27 172 (23.03)	175 789 (38.91)
Physical activity (1–4)	1.89 (0.78)	2.02 (0.80)	2.12 (0.80)	2.02 (0.80)
Body mass index (kg/m^2^)	25.77 (4.19)	25.38 (3.78)	24.77 (3.44)	25.30 (3.80)
Total cholesterol (mmol/L)	5.88 (1.17)	5.69 (1.08)	5.48 (1.06)	5.67 (1.10)
Systolic blood pressure (mm Hg)	131.76 (17.14)	130.09 (15.45)	127.30 (14.84)	129.72 (15.75)

Note: Presented as mean (standard deviationSD) or count (percentages).

CVDcardiovascular disease

Table 2 shows the results from the models adjusted for sex and one of the risk factors separately using a parametric survival model (Weibull model). The estimated HRs associated with current smoking status were 1.34 (95% CI 1.27 to 1.43) in the low education group, 1.88 (95% CI 1.79 to 1.98) in the middle education group and 2.22 (95% CI 2.00 to 2.46) in the high education group (table 2). The mutually adjusted estimates were attenuated.

**Table 2:** HR and 95% CI for CVD mortality according to established risk factors for CVD among participants in Norwegian health surveys aged 35–65 years at attendance (N=451 800), estimated using proportional hazards model with Weibull baseline and stratified by educational level

	Educational level			All
	Low	Middle	High	
Model 1				
Current smoking (yes)	1.34 (1.27 to 1.43)	1.88 (1.79 to 1.98)	2.22 (2.00 to 2.46)	2.02 (1.94 to 2.09)
Physical activity	0.79 (0.76 to 0.82)	0.79 (0.76 to 0.81)	0.86 (0.81 to 0.91)	0.75 (0.74 to 0. 77)
Body mass index	1.06 (1.06 to 1.07)	1.06 (1.06 to 1.07)	1.08 (1.07 to 1.09)	1.08 (1.08 to 1.08)
Total cholesterol	1.43 (1.41 to 1.45)	1.31 (1.30 to 1.32)	1.08 (1.07 to 1.09)	1.09 (1.09 to 1.10)
Systolic blood pressure	1.03 (1.03 to 1.04)	1.04 (1.03 to 1.04)	1.04 (1.04 to 1.04)	1.04 (1.04 to 1.04)
Model 2				
Current smoking (yes)	1.55 (1.46 to 1.65)	1.98 (1.88 to 2.09)	2.23 (2.01 to 2.48)	2.13 (2.06 to 2.21)
Physical activity	0.86 (0.83 to 0.90)	0.87 (0.84 to 0.89)	0.93 (0.87 to 0.99)	0.83 (0.81 to 0.85)
Body mass index	1.02 (1.02 to 1.03)	1.02 (1.02 to 1.03)	1.04 (1.02 to 1.05)	1.04 (1.03 to 1.04)
Total cholesterol	1.33 (1.31 to 1.36)	1.22 (1.21 to 1.24)	1.08 (1.07 to 1.09)	1.10 (1.10 to 1.10)
Systolic blood pressure	1.03 (1.03 to 1.03)	1.03 (1.03 to 1.03)	1.04 (1.03 to 1.04)	1.04 (1.03 to 1.04)

Model 1: adjusted for sex. Model 2: adjusted for sex and the other CVD risk factors (current smoking, physical activity, body mass index, total cholesterol, systolic blood pressure). Numbers in parentheses are the corresponding 95% CI.

CVDcardiovascular mortality

We then investigated how the estimated variance of frailty distribution varied in the models without the risk factors. The estimated frailty variance parameter θ increased from 24.96 (95% CI 20.79 to 29.97) among those in the lowest to 34.12 (95% CI 28.09 to 41.44) in the middle and 42.37 (95% CI 25.67 to 69.93) in the highest education group (table 3).

**Table 3:** Estimated HR for CVD mortality according to educational level from the models without the risk factors with Weibull baseline hazard distribution and gamma frailty distribution

	Educational level			All
	Low	Middle	High	
Sex (male)	3.56 (3.10 to 4.07)	4.07 (3.63 to 4.58)	4.66 (3.73 to 5.83)	3.58 (3.31 to 3.89)
Log (constant)	−29.92 (−31.67 to −28.18)	−30.90 (−32.38 to −29.41)	−31.66 (−34.49 to −28.83)	−29.83 (−30.87 to −28.80)
$\hat{p}$	6.61 (6.17 to 7.07)	6.55 (6.19 to 6.93)	6.50 (5.84 to 7.25)	6.35 (6.10 to 6.62)
$\overset{⌢}{\theta }$	24.96 (20.79 to 29.97)	34.12 (28.09 to 41.44)	42.37 (25.67 to 69.93)	33.42 (29.15 to 38.32)
${\bar{\chi }}^{2}$ (p value)	154 (<0.001)	88 (<0.001)	17 (<0.001)	244 (<0.001)
AIC	30 831	44 745	13 312	91 945

Numbers in parentheses are the corresponding 95% CI. ${\bar{\chi }}^{2}$is the statistics for the likelihood ratio test of H_0_ θ=0. $\hat{p}$ is the estimated shape parameter that enables the density function to adapt into various shapes, facilitating effective data fitting. $\overset{⌢}{\theta }$ is the estimated gamma parameter.

AICAkaike’s information criterionCVDcardiovascular disease

Table 4 presents the frailty analysis adjusting for risk factors. Men had a higher risk of CVD mortality compared with women in all levels of education. Physical activity had low HRs (less than 1) in all levels of education. The results for body mass index, total cholesterol and systolic blood were consistent with those from the models without frailty in table 2. HRs associated with current smoking status in table 2 were slightly smaller than those in table 4. In tables2  4, HRs associated with total cholesterol decreased from low to high level; however, those in table 2 were slightly smaller than those in table 4. As shown in table 4, the estimated frailty variance θ increased from 3.76 (95% CI 2.83 to 4.99) among those in the lowest, to 7.12 (95% CI 5.51 to 9.19) in the middle and to 7.82 (95% CI 4.42 to 13.85) in the highest education group. All frailty variances were significantly different from 0, and the frailty models were preferred over the non-frailty models in terms of Akaike’s information criterion. However, as seen in tables3  4, the estimated parameters of the Weibull baseline hazard are quite similar across educational groups (with largely overlapping 95% CIs, especially for parameter p). Thus, the differences in the frailty variances across educational groups could represent actual differences in the underlying individual heterogeneity across these groups. The sensitivity analysis was consistent with the main analysis (online supplemental table 2).

**Table 4:** HR and 95% CI for CVD mortality according to established risk factors for CVD among participants in Norwegian health surveys aged 35–65 years at attendance (N=451 800), estimated with Weibull baseline hazard distribution and gamma frailty distribution using age at survey and stratified by educational level

	Educational level			All
	Low	Middle	High	
Sex (male)	1.80 (1.68 to 1.95)	2.07 (1.92 to 2.22)	2.13 (1.86 to 2.43)	1.80 (1.72 to 1.89)
Current smoking (yes)	1.67 (1.55 to 1.79)	2.26 (2.11 to 2.42)	2.39 (2.10 to 2.72)	2.30 (2.19 to 2.41)
Physical activity	0.84 (0.80 to 0.88)	0.84 (0.81 to 0.88)	0.94* (0.88 to 1.01)	0.83 (0.81 to 0.85)
Body mass index	1.03 (1.02 to 1.03)	1.03 (1.02 to 1.03)	1.03 (1.01 to 1.05)	1.03 (1.03 to 1.04)
Total cholesterol	1.45 (1.41 to 1.50)	1.41 (1.37 to 1.45)	1.36 (1.29 to 1.44)	1.46 (1.43 to 1.49)
Systolic blood pressure	1.04 (1.04 to 1.04)	1.04 (1.04 to 1.04)	1.04 (1.04 to 1.04)	1.04 (1.04 to 1.04)
Log (constant)	−33.0 (−34.2 to −31.7)	−35.4 (−36.6 to −34.3)	−36.6 (−38.6 to −34.6)	−34.9 (−35.6 to −34.1)
$\hat{p}$	5.31 (5.08 to 5.55)	5.68 (5.46 to 5.91)	5.85 (5.45 to 6.27)	5.48 (5.34 to 5.63)
$\overset{⌢}{\theta }$	3.76 (2.83 to 4.99)	7.12 (5.51 to 9.19)	7.82 (4.42 to 13.85)	5.45 (4.58 to 6.50)
${\bar{\chi }}^{2}$ (p value)	100 (<0.001)	226 (<0.001)	88 (<0.001)	1156 (<0.001)
AIC†	27 640	40 983	12 325	82 207
AIC‡	27 738	41 207	12 411	83 361

Numbers in parentheses are the corresponding 95% CI. ${\bar{\chi }}^{2}$ is the likelihood ratio test statistics of H_0_ θ=0. $\hat{P}$ is the estimated shape parameter that enables the density function to adapt into various shapes, facilitating effective data fitting. $\overset{⌢}{\theta }$ is the estimated gamma parameter.

* Indicates that the corresponding covariate is not statistically significant at 5%.

† AIC value of the frailty models.

‡ AIC value of the non-frailty models.

AICAkaike’s information criterionCVDcardiovascular mortality

**Figure 1:**
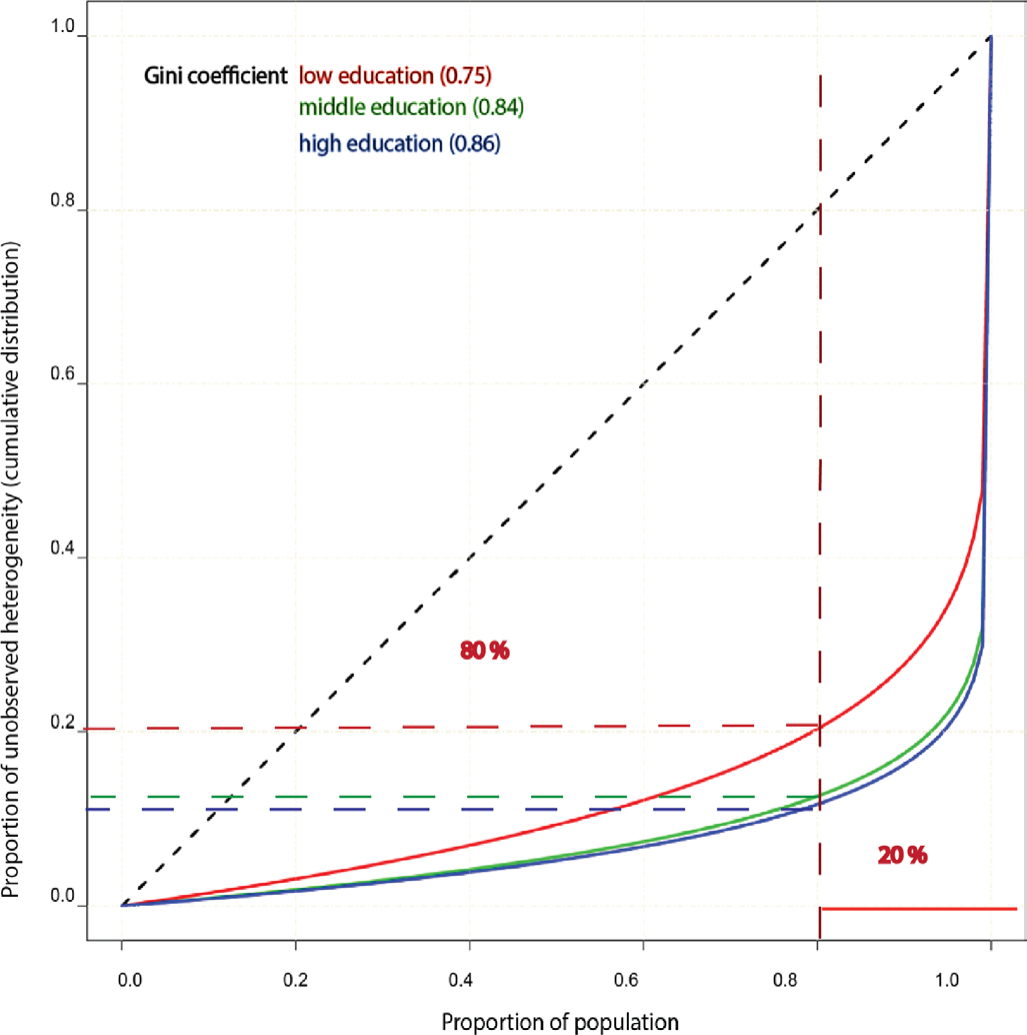
Lorenz curves and Gini coefficients are displayed for three educational levels using Norwegian pooled health surveys. Gini coefficient is a summary measure of variation in individual risk. Lorenz curve provides a visual description about the inequality in individual risk.

Figure 1 provides a graphical representation of inequalities in individual risk according to the three levels of education, estimated as Gini coefficients, with the coefficients being 0.75, 0.84 and 0.86 for the low, middle and high education group, respectively. Although the differences between the frailty variances are not very large and the 95% CIs of the high education group largely overlap the two other groups, a tendency of increased variation from the low to the high education group is seen. This trend was visually depicted by the Lorenz curves, highlighting the clear difference in distribution of unobserved heterogeneity across educational levels. We also performed additional frailty analysis for the population without stratification where education was treated as a categorical covariate in addition to the risk factors in table 4. The estimated frailty variance θ then changed from 5.45 (95 % CI 5.34 to 5.63) to 5.14 (95% CI 4.32 to 6.11). This was consistent with the results from the stratified analysis that frailty variances were significantly different from 0.

## Discussion

### Main findings

In a sample based on data from participants in Norwegian cardiovascular health surveys, we found that the unexplained variation in the risk of dying from CVD, which may also be referred to as individual heterogeneity, was substantial even after adjusting for established risk factors. Our results also indicate that unobserved heterogeneity in CVD could be somewhat higher in participants with high educational attainment compared with those with low educational attainment.

### Methodological considerations and limitations

Compared with a typical survival model, additional variation from unobserved factors was taken into account and estimated as a separate parameter in our models. Even though the HR estimates were similar in direction for all the risk factors in the models with and without frailty, there remained some differences in the estimates. It was not the goal of this paper to assess the causal contribution of each CVD risk factor. Various approaches have been used to characterise individual risk of CVD. The Framingham risk score and other risk calculators are well known and commonly used in clinical practice.^23^ However, the performance of these models based on, among others, the c-statistics of the receiver operating curve is only able to account for the observed risk factors and does not explicitly model the unobserved part.^24^ Our method accounted for both observed and unobserved risk factors. In contrast to models aiming to predict the risk of future events in individuals, our main concern here was to assess the role of the remaining individual variation at the population level.

Our final study population may differ from the general population due to different participation rates in the health surveys. The participants excluded for missing values tended to be older (online supplemental table 1) and more likely to have died during the follow-up period than the average participants. Additionally, self-reported information on current smoking status did not contain information on the frequency of smoking. We chose to include participants 35–65 years of age at survey to avoid pooling data from surveys where the start of follow-up was very different in calendar time (1974–2002). The age of 35 was chosen as the start of follow-up since the incidence and prevalence of CVD increase with advancing age from 35.^25^ Our survival time was converted to years.

We used parametric survival models that assume a specific distribution for the survival time. A crucial assumption in our analysis is the assumption of a Weibull baseline hazard rate indicating that the individual risk of CVD mortality has a Weibull form. This is an untestable assumption, but we argue that it is reasonable.^26^ The choice of Weibull distribution is based on the underlying biological process relating to CVD. The individual CVD risk level is developing over time due to changes that occur in the cardiovascular system with age, although the risk estimates for risk factors may abate in the oldest age groups.^27^ Some CVD risk factors are also associated with the onset of advanced age, for instance, obesity and diabetes.^27^ Ageing has a crucial effect on the heart and arterial system, leading to increased risk for CVDs, including atherosclerosis, hypertension, myocardial infarction and stroke.^28^ The heart thickens and stiffens with age, leading to increased imposition of a number of functional demands. There is increased incidence of disease, a less structurally efficient heart and decreased cardiac reserve associated with ageing.^29^ For example, it has been shown that the carotid wall intima media thickness increases twofold to threefold between 20 and 90 years of age.^28^

Part of the frailty variance might be due to the omission of relevant covariates such as diabetes and alcohol consumption. We did not control for diabetes due to the significant number of missing values for diabetes and because it was self-reported. Excessive alcohol use was only available for a smaller proportion. Also, selection bias could arise because age variability differed between educational groups. When analysing the A40P participants, the results were consistent. Additionally, this study did not fully address the impact of confounding factors such as generational differences, lifestyle and the interplay of social factors like race, gender, socioeconomic status and age. However, another study on birth cohorts pre-1945 and post-1945 showed a decline in premature mortality, yet the variation in CVD mortality remained consistent across these cohorts. We analysed both sexes jointly by adjusting for sex, and we were not able to assess if the educational variances in CVD mortality differ by sex. Adding sex into the model did not change the results. The use of the gamma distribution in the analysis of bivariate survival data, like CVD mortality, is preferred for its advantageous properties. It offers a more effective statistical analysis for understanding and interpreting CVD mortality.^30^,^32^

Another limitation may be that we fitted models to each educational group separately. This means that the frailty variances in the three educational groups may not be directly comparable because separate baseline hazards are estimated. However, given the similarity of the Weibull parameters in the baseline hazard and that the estimated model coefficients of the CVD risk factors did not change substantially, we have interpreted the differences in the frailty variance as indication of differences between the educational groups. Furthermore, our sensitivity analysis in the unstratified model was consistent with this, showing some attenuation after adjusting for education but with substantial remaining variation.

### Interpretation

There has previously been a substantial interest in assessing to what extent established risk factors explain social inequalities in CVD as this has important implications for prevention of these inequalities.^9^ Evidence seems to suggest that the modifiable cardiometabolic and health behavioural risk factors explain a major share of the inequalities in the population, especially if they are measured repeatedly and using absolute measures of differences between social groups.^9^ Our study supports this using a different approach. The Gini coefficients are large compared with many cancers.^33^ The Lorentz curve shows that the 20% individuals with the highest risk account for around 80%–87% of the variation in individual risk.^34^ This is consistent with recent evidence using polygenic risk scores which suggests that in the population there might be a subgroup with very high risk even after traditional risk factors and education are taken into account.^35^

At the general level, several explanations for the differences in CVD risk between individuals may be relevant. First is the gene–environment interaction for the established risk factors with stronger causal effects in subgroups.^12^ Second is that cognitive ability and other latent unmeasured personal factors related to education may put individuals repeatedly at risk through unhealthy behaviour, environment and lifestyle. Measurement of risk factors only at baseline may here be insufficient in capturing risk. Some of these personal factors could have genetic origin and be related to the environment and lifestyle from infancy through the life course by gene–environment correlation.^5 32^ Third, early life factors may play a role, including both non-genetic factor and potential interactions with genetics. Shared environmental factors in early life may be transmitted from the family environment or intrauterine exposures. Other potential risk factors for CVD have been suggested, such as ankle-brachial index, high-sensitive C reactive protein level and coronary artery calcium score. Their contribution, in addition to established risk factors, to risk prediction is considered modest.^36^ Finally, these differences could be a result of random events adding up risk over the life course. Many of these are often difficult to measure and observe, but they need to follow a non-random pattern in order to play a role in social inequalities. Unfortunately, we were not able to study cognitive ability here as the sample size was inadequate. Studies of cognitive ability and later health have proposed several explanations for the association. High cognitive ability may include better health literacy and uptake of healthy behaviour. However, it could also give people a general tendency related to health, which is difficult to measure with risk factors.^8^ Here we stratified by education and found larger variation in the high educated groups. This can arise as a consequence of the relative importance of risk factors compared with other unobserved factors. Lower educated groups have higher levels of risk factors such as smoking. Hence, in the low educated group, these probably dominate the risk, whereas in the high educated other factors may become proportionally more important. This is consistent with the point made by Geoffrey Rose that if everyone in a population smokes, then social differences in lung cancer are proportionately more explained by other factors such as asbestos exposure.^37^ Interventions to reduce tobacco smoking, such as increased taxes on alcohol and tobacco, would still have a significantly stronger impact on reducing absolute inequalities.^9^ Our results should be further investigated in available samples with relevant risk factors. We also acknowledge that model misspecification could be attributed to the frailty in the type of analyses performed here. Underlying heterogeneity may differ between women and men.^38^,^40^ Future research should investigate this.

## Conclusion

Even if a large share of the variation in the risk of dying from CVD was explained by the risk factors, we found a substantial remaining variation. Furthermore, our results indicate that there could be a somewhat larger unobserved heterogeneity in the risk in groups with higher education. Our findings indicate that the unobserved variation in CVD mortality could vary somewhat between educational groups but that education does not explain a major share of the remaining substantial heterogeneity.

## supplementary material

10.1136/bmjph-2023-000104 online supplemental file 1

## Data Availability

No data are available.

## References

[1] Ezzati M, Obermeyer Z, Tzoulaki I (2015). Contributions of risk factors and medical care to cardiovascular mortality trends. Nat Rev Cardiol.

[2] Yusuf S, Hawken S, Ounpuu S (2004). Effect of potentially modifiable risk factors associated with myocardial infarction in 52 countries (the INTERHEART study): case-control study. Lancet.

[3] Popham F, Dibben C, Bambra C (2013). Are health inequalities really not the smallest in the Nordic welfare states? A comparison of mortality inequality in 37 countries. J Epidemiol Community Health.

[4] Mackenbach JP (2012). The persistence of health inequalities in modern welfare states: the explanation of a paradox. Soc Sci Med.

[5] Mackenbach JP (2010). New trends in health inequalities research: now it’s personal. Lancet.

[6] Plomin R, Deary IJ (2015). Genetics and intelligence differences: five special findings. Mol Psychiatry.

[7] Bijwaard GE, Tynelius P, Myrskylä M (2019). Education, cognitive ability, and cause-specific mortality: A structural approach. Popul Stud (Camb).

[8] Batty GD, Deary IJ, Gottfredson LS (2007). Premorbid (early life) IQ and later mortality risk: systematic review. Ann Epidemiol.

[9] Lynch J, Davey Smith G, Harper S (2006). Explaining the social gradient in coronary heart disease: comparing relative and absolute risk approaches. J Epidemiol Community Health.

[10] Singh-Manoux A, Nabi H, Shipley M (2008). The role of conventional risk factors in explaining social inequalities in coronary heart disease: the relative and absolute approaches to risk. Epidemiology.

[11] Ariansen I, Graff-Iversen S, Stigum H (2015). Do repeated risk factor measurements influence the impact of education on cardiovascular mortality?. Heart.

[12] Aalen OO, Valberg M, Grotmol T (2015). Understanding variation in disease risk: the elusive concept of frailty. Int J Epidemiol.

[13] Vaupel JW, Manton KG, Stallard E (1979). The impact of heterogeneity in individual frailty on the dynamics of mortality. Demography.

[14] Balan TA, Putter H (2020). A tutorial on frailty models. Stat Methods Med Res.

[15] Rubio FJ, Putter H, Belot A (2023). Individual frailty excess hazard models in cancer epidemiology. Stat Med.

[16] Naess O, Søgaard AJ, Arnesen E (2008). Cohort profile: cohort of Norway (CONOR). Int J Epidemiol.

[17] Tverdal A, Selmer RM (2002). Screening of 40-year-olds--400,000 men and women attended. Tidsskr Nor Laegeforen.

[18] Bjartveit K, Foss OP, Gjervig T (1979). The cardiovascular disease study in Norwegian counties. Background and organization. Acta Med Scand Suppl.

[19] Ibsen E (1994). Educational changes in Norway. J Educ Teach.

[20] Kızılersü A, Kreer M, Thomas AW (2018). The weibull distribution. Signif Oxf.

[21] Gutierrez RG (2002). Parametric frailty and shared frailty survival models. Stata J.

[22] Cowell F, Atkinson AB, Bourguignon F (2000). Handbook of income distribution.

[23] Lloyd-Jones DM, Wilson PWF, Larson MG (2004). Framingham risk score and prediction of lifetime risk for coronary heart disease. Am J Cardiol.

[24] Caetano SJ, Sonpavde G, Pond GR (2018). C-statistic: A brief explanation of its construction, interpretation and limitations. Eur J Cancer.

[25] Govender RD, Al-Shamsi S, Soteriades ES (2019). Incidence and risk factors for recurrent cardiovascular disease in middle-eastern adults: a retrospective study. BMC Cardiovasc Disord.

[26] Ip EH, Efendi A, Molenberghs G (2015). Comparison of risks of cardiovascular events in the elderly using standard survival analysis and multiple-events and recurrent-events methods. BMC Med Res Methodol.

[27] Savarese G, Gotto AM, Paolillo S (2013). Benefits of statins in elderly subjects without established cardiovascular disease: a meta-analysis. J Am Coll Cardiol.

[28] Lakatta EG, Levy D (2003). Arterial and cardiac aging: major shareholders in cardiovascular disease enterprises: Part I: aging arteries: a “set up” for vascular disease. Circulation.

[29] Strait JB, Lakatta EG (2012). Aging-associated cardiovascular changes and their relationship to heart failure. Heart Fail Clin.

[30] Shih JH, Louis TA (1995). Assessing gamma frailty models for clustered failure time data. Lifetime Data Anal.

[31] Hanagal DD, Rao ASRS, Pyne S, Rao CR (2017). Disease modelling and public health, part B.

[32] Khera AV, Emdin CA, Drake I (2016). Genetic Risk, Adherence to a Healthy Lifestyle, and Coronary Disease. N Engl J Med.

[33] Stensrud MJ, Valberg M (2017). Inequality in genetic cancer risk suggests bad genes rather than bad luck. Nat Commun.

[34] Mauguen A, Begg CB (2016). Using the Lorenz Curve to Characterize Risk Predictiveness and Etiologic Heterogeneity. Epidemiology.

[35] Khera AV, Chaffin M, Aragam KG (2018). Genome-wide polygenic scores for common diseases identify individuals with risk equivalent to monogenic mutations. Nat Genet.

[36] Curry SJ, Krist AH, US Preventive Services Task Force (2018). Risk Assessment for Cardiovascular Disease With Nontraditional Risk Factors: US Preventive Services Task Force Recommendation Statement. JAMA.

[37] Rose G (2001). Sick individuals and sick populations. Int J Epidemiol.

[38] Simandan D (2019). Revisiting positionality and the thesis of situated knowledge. Dialogues Hum Geogr.

[39] Simandan D (2021). Social capital, population health, and the gendered statistics of cardiovascular and all-cause mortality. SSM Popul Health.

[40] Wilson Y, White A, Jefferson A (2019). Intersectionality in Clinical Medicine: The Need for a Conceptual Framework. Am J Bioeth.

