# Supplementary material for: Individual heterogeneity, educational attainment and cardiovascular mortality: a pooled analysis of Norwegian health surveys
Source: BMJ Public Health. 2024 Sep 23;2(2):e000104. doi: 10.1136/bmjph-2023-000104 (PMC11816836; doi:10.1136/bmjph-2023-000104)
Supplement: online supplemental file 1 [file bmjph-2-2-s001.pdf]

Supplementary Material

Individual heterogeneity, educational attainmen and cardiovascular mortality - A pooled analysis of Norwegian health surveys

Huong Nguyen Thu[1,5], Tron Anders Moger[2], Morten Valberg[1,3], Eirik Degerud[4], Christian M. Page[5,6], Marissa LeBlanc[7,8], Øyvind Næss[1,9]

1. Department of Community Medicine and Global Health, University of Oslo, Oslo, Norway
2. Department of Health Management and Health Economics, University of Oslo, Oslo, Norway
3. Oslo Centre for Biostatistics and Epidemiology, Oslo University Hospital, Oslo, Norway
4. National Institute of Occupational Health (STAMI), PO Box 5330 Majorstuen, N-0033, Oslo
5. Centre for Fertility and Health, Norwegian Institute of Public Health, Oslo, Norway
6. Section for Statistics, Department of Mathematics, University of Oslo, Oslo, Norway
7. Division of Infection Control, Norwegian Institute of Public Health, Oslo, Norway
8. Oslo Centre for Biostatistics and Epidemiology, University of Oslo, Oslo, Norway
9. Norwegian Institute of Public Health, Oslo, Norway

*** Correspondence:** Huong Nguyen Thu, PhD: HuongThu.Nguyen@fhi.no

1 Supplementary Figure and Tables

1.1 Supplementary Figure

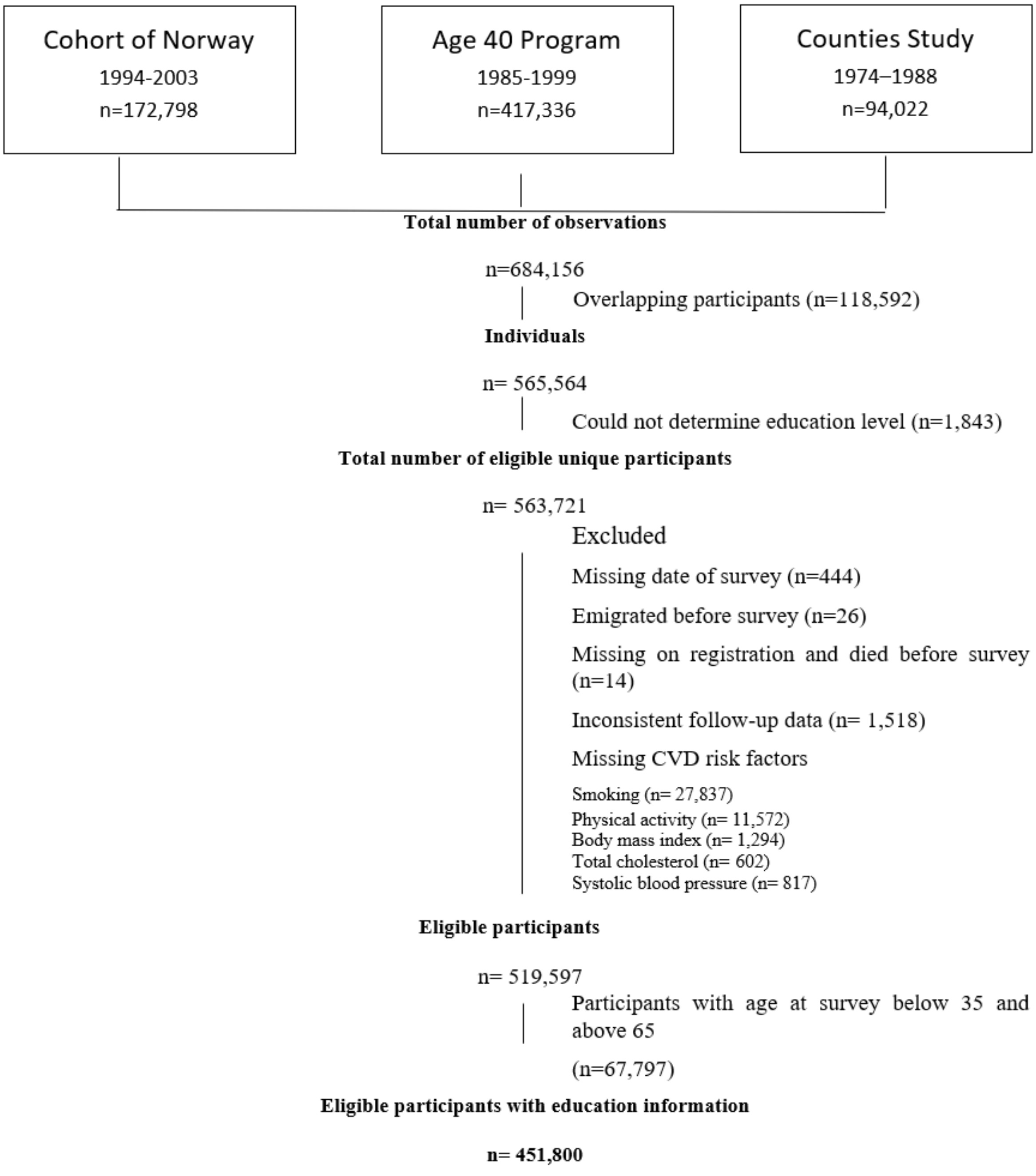

Supplementary Figure 1. Flow chart on inclusion and exclusion of the study population.

1.2 Supplementary Tables

Supplementary Table 1. Descriptive statistics of individuals excluded from the study population because of missing values.

Variable	Source population	Excluded for missing values		Study population
		**Education**	**CVD risk factors**	
	**(n = 565564)**	**(n=1843)**	**(n=42585)**	**(n=451800)**
Age	44.0 (10.0)	44.4 (11.8)	46.8 (13.1)	42.6 (5.3)
Sex (male), n (%)	272093 (48.1)	939 (51.1)	20198 (47.4)	215857 (47.8)
CVD deaths, n (%)	27164 (4.8)	104 (5.6)	4107 (9.6)	11932 (2.6)

Note: Individuals with missing data on education and data on CVD risk factors were not described here. Age presented as mean (standard deviation). Abbreviations: CVD, cardiovascular disease.

Supplementary Table 2. Hazard ratios and 95% CIs for CVD mortality according to established risk factors of CVD among participants in the Age 40 Program aged 35 to 65 years at attendance (n = 373,808), estimated with Weibull baseline hazard distribution and Gamma frailty distribution using age at survey, and stratified by educational level.

	**Education levels**			
	Low	Middle	High	All
Sex (male)	1.55 (1.68 – 1.95)	1.83 (1.69 – 1.99)	2.01 (1.72 – 2.36)	1.56 (1.48 – 1.65)
Current smoking (yes)	1.44 (1.55 – 1.79)	2.23 (2.07 – 2.41)	2.45 (2.11 – 2.84)	2.19 (2.08 – 2.30)
Physical activity	0.86 (0.80 – 0.88)	0.88 (0.84 – 0.92)	0.95* (0.88 – 1.04)	0.86 (0.83 – 0.89)
Body mass index	1.02 (1.02 – 1.03)	1.02 (1.01 – 1.03)	1.01* (0.99 – 1.03)	1.02 (1.02 – 1.03)
Total cholesterol	1.51 (1.41 – 1.50)	1.46 (1.41 – 1.51)	1.41 (1.32 – 1.50)	1.52 (1.48 – 1.56)
Systolic blood pressure	1.04 (1.04 – 1.04)	1.04 (1.04 – 1.05)	1.04 (1.04 – 1.05)	1.05 (1.04 – 1.04)
Log(constant)	-32.7 (-34.1 – -31.3)	-34.6 (-35.9 – -33.3)	-34.9 (-37.2 – -32.5)	-36.6 (-38.6 – -34.6)
$\hat{p}$	5.07 (4.82 – 5.33)	5.37 (5.13 – 5.63)	5.40 (4.95 – 5.89)	5.17 (5.01 – 5.33)
$\hat{\theta}$	3.70 (2.73 – 5.01)	7.92 (5.94 – 10.56)	8.06 (3.82 – 16.98)	5.72 (4.70 – 6.97)
$\bar{\chi}^2$ (p-value)	118 (<0.001)	92.43 (<0.001)	88 (<0.001)	88 (<0.001)

Note: Numbers in parentheses are the corresponding 95% confidence intervals. $\bar{\chi}^2$ is the likelihood-ratio (LR) test statistic of H_0 θ = 0. The notation * indicates that the corresponding covariate is not statistically significant at 5%. $\hat{p}$: estimated shape parameter that enables the density function to adapt into various shapes, facilitating effective data fitting. $\hat{\theta}$: estimated Gamma parameter.
Abbreviations: CVD, cardiovascular mortality; HR, hazard ratio; CI, confidence interval.
